# Gut Microbiome Alterations in Colorectal Cancer: Mechanisms, Therapeutic Strategies, and Precision Oncology Perspectives

**DOI:** 10.3390/cancers17142294

**Published:** 2025-07-10

**Authors:** Miriam Tudorache, Andreea-Ramona Treteanu, Gratiela Gradisteanu Pircalabioru, Irina-Oana Lixandru-Petre, Alexandra Bolocan, Octavian Andronic

**Affiliations:** 1 Faculty of Medicine, Carol Davila University of Medicine and Pharmacy Bucharest, 050474 Bucharest, Romania; miriam.tudorache0720@stud.umfcd.ro (M.T.); andreea-ramona.treteanu0720@stud.umfcd.ro (A.-R.T.); 2 Innovation and eHealth Center, Carol Davila University of Medicine and Pharmacy Bucharest, 010451 Bucharest, Romania; octavian.andronic@umfcd.ro; 3 Faculty of Biology, University of Bucharest, 050095 Bucharest, Romania; 4 Research Institute of University of Bucharest (ICUB), University of Bucharest, 050663 Bucharest, Romania; 5 eBio-Hub Centre of Excellence in Bioengineering, National University of Science and Technology Politehnica Bucharest, 060042 Bucharest, Romania; irina.petre@upb.ro; 6 General Surgery Department, Carol Davila University of Medicine and Pharmacy Bucharest, 050474 Bucharest, Romania; alexandra.bolocan@umfcd.ro

**Keywords:** colorectal cancer, microbiome modulation, microbiome alteration, therapeutic strategies, personalized oncology

## Abstract

Colorectal cancer (CRC) is one of the most prevalent and lethal oncological diseases worldwide, with a concerning rise in incidence, particularly in developing countries. Recent advances in genetic sequencing have revealed that the gut microbiome plays a crucial role in CRC development. Mechanisms such as chronic inflammation, metabolic alterations, and oncogenic pathways have demonstrated that dysbiosis, a disruption of the gut microbiome, is linked to CRC. Associations have been found between tumor progression, treatment resistance, and pathogenic microbes such as *Fusobacterium nucleatum* and *Escherichia coli*. A promising approach for CRC prevention and treatment is microbiome manipulation through interventions such as probiotics, prebiotics, fecal microbiota transplantation, and selective antibiotics. This article explores how gut microbiome alterations influence CRC pathogenesis and examines microbiome modulation strategies currently used as adjuncts to traditional treatments. Advances in artificial intelligence, single-cell and spatial transcriptomics, and large-scale initiatives such as the ONCOBIOME Project are paving the way for the identification of microbiome-derived biomarkers for early CRC detection and personalized treatment. Despite promising progress, challenges such as interindividual variability, causal inference, and regulatory hurdles must be addressed. Future integration of microbiome analysis into multi-omics frameworks holds great potential to revolutionize precision oncology in CRC management.

## Simple Summary

Colorectal cancer (CRC) is a leading cause of cancer-related deaths worldwide. Recent research shows that the gut microbiome—microorganisms living in our intestines—plays a crucial role in CRC development. An imbalance in these microbes, known as dysbiosis, can promote inflammation, produce harmful substances, and disrupt normal cell functions, leading to cancer. Specific bacteria like *Fusobacterium nucleatum* and *Escherichia coli* have been linked to tumor growth and resistance to treatment. Promising therapeutic strategies include probiotics, prebiotics, fecal microbiota transplantation, dietary changes, and selective antibiotics. Advances in technologies such as artificial intelligence and microbiome sequencing are helping identify microbial biomarkers for early detection and personalized treatment. Despite the potential, challenges remain, including individual variability and the need for standardized clinical methods. This review highlights the growing importance of gut microbiota in CRC prevention, diagnosis, and therapy, aiming to support the development of microbiome-targeted strategies in personalized oncology.

## 1. Introduction

Colorectal cancer (CRC) is widely acknowledged as one of the most common malignancies on a global scale and continues to constitute a major cause of cancer-associated morbidity and mortality [1,2,3]. According to GLOBOCAN 2020 estimates, CRC is the third most frequently diagnosed malignancy and the second leading cause of cancer-related death, with over 1.9 million new cases and approximately 930,000 deaths annually [4]. As the global population continues to age and more communities adopt a Western lifestyle, there is a concerning trend of increasing CRC incidence among young individuals, particularly in developing countries [2,3,5,6,7,8]. These statistics highlight the urgent need for innovative therapeutic approaches to improve patient prognosis and quality of life.

In recent years, advancements in genetic sequencing technology have facilitated the exploration of the gut microbiome and its role in CRC pathogenesis [9,10]. Intestinal dysbiosis—characterized by alterations in the composition and function of the gut microbiota—has been shown to contribute to colorectal carcinogenesis through multiple mechanisms, including chronic inflammation, metabolic changes, and the modulation of oncogenic pathways [11,12,13,14,15]. Certain pathogenic agents, such as *Fusobacterium nucleatum* and colibactin-producing *Escherichia coli*, have been associated with tumor progression and treatment resistance [12,16,17]. Additionally, bioactive metabolites like short-chain fatty acids (SCFAs), secondary bile acids derived from the microbiota, and other polygenic factors can significantly impact CRC response and outcomes [18,19]. As a result of these recent findings, gut microbiota modulation has emerged as a promising strategy for CRC prevention and treatment, offering potential improvements in patient prognosis and quality of life [12,13].

The significance of this line of research stems from the promising therapeutic implications of targeted microbiota modulation as an adjunctive approach for both the prevention and management of colorectal cancer. Specific interventions, including the administration of defined probiotic strains, supplementation with selective prebiotics, fecal microbiota transplantation (FMT), and the judicious use of narrow-spectrum antibiotics, have the potential to restore a beneficial microbial composition. Such strategies may enhance mucosal immunity, mitigate pro-tumorigenic inflammation, and ultimately improve patients’ responses to conventional oncologic therapies. The aim of this review article was to analyze the impact of gut microbiome alterations on CRC development and progression and to evaluate current and emerging interventions for microbiota modulation as a therapeutic strategy. The proposed objectives include identifying the key mechanisms by which the microbiome influences colorectal carcinogenesis, assessing the current evidence on microbiome-based therapy, and discussing the challenges and future directions in this emerging field of personalized oncology.

## 2. Intestinal Dysbiosis and Colorectal Carcinogenesis

### 2.1. Advancements in Microbiome Research and CRC

Advancements in sequencing technologies have significantly enhanced our ability to conduct large-scale investigations into microbiome alterations present in various human diseases, including colorectal cancer (CRC) [9,12,20,21]. The homeostasis of the intestinal microbiota is essential for maintaining overall host health and well-being [22]. However, even a minor disruption in this delicate balance—referred to as dysbiosis—is directly linked to colorectal carcinogenesis, raising multiple concerns regarding this disease [9,13].

A healthy microbiome maintains equilibrium between commensal and opportunistic bacteria. However, various factors such as an imbalanced diet, frequent antibiotic use, chronic inflammation, and genetic predisposition can disrupt this balance, leading to dysbiosis [20,23]. In CRC patients, the gut microbiome undergoes significant changes characterized by reduced bacterial diversity and the proliferation of pathogenic species [24]. Among the microorganisms most frequently associated with CRC are *Fusobacterium nucleatum*, pathogenic *Escherichia coli*, and *Bacteroides fragilis*, which have been implicated in inflammatory processes and cellular homeostasis disruption [16,17,23].

### 2.2. Chronic Inflammation and Immune Modulation

One of the key mechanisms through which dysbiosis contributes to CRC is the induction of chronic inflammation, which leads to an altered immune response and promotes a favorable environment for uncontrolled cell proliferation [9,11,18,25]. *Fusobacterium nucleatum* has been identified as a driver of intestinal inflammation and tumor progression by interacting with immune cells and inhibiting the antitumor immune response [26,27,28]. Pathogenic *Escherichia coli* produces colibactin, a genotoxin capable of inducing DNA damage and genomic instability, thereby accelerating oncogenic processes [28]. Additionally, *Bacteroides fragilis* secretes toxins that disrupt intestinal barrier integrity and stimulate inflammatory pathways, creating a microenvironment conducive to tumor development [19].

### 2.3. Production of Toxic Metabolites

Beyond inflammation, the gut microbiota contributes to CRC through the production of toxic metabolites. Colibactin produced by *E. coli* induces genetic mutations and oxidative stress, promoting the malignant transformation of intestinal epithelial cells [20]. Secondary bile acids, generated from fat metabolism by certain bacterial species, exhibit pro-inflammatory and pro-carcinogenic effects, directly damaging DNA integrity and stimulating cell proliferation [18]. Butyrate, a metabolite produced by fermentative bacteria, plays a paradoxical role: under normal conditions, it exerts anti-inflammatory and pro-apoptotic effects beneficial for colon health [29]. However, in a dysbiotic state, its concentration may become unfavorable, negatively impacting epithelial cell metabolism [30].

### 2.4. Genetic and Epigenetic Alterations in CRC

Colorectal cancer is a complex and heterogeneous disease caused by the cumulative acquisition of not only genetic mutations but also widespread epigenetic modifications over time [6]. These alterations lead to the dysregulation of numerous pathways essential for maintaining normal cellular function and homeostasis [6]. The intricate interplay between genetic and epigenetic changes plays a fundamental role in the transition from benign adenomatous polyps to malignant CRC [6,9].

### 2.5. Microbiota Interaction with Oncogenic Pathways

The intestinal microbiota interacts with critical oncogenic pathways in CRC, such as p53 and Wnt/β-catenin [28]. *Fusobacterium nucleatum* has been shown to interfere with the Wnt/β-catenin pathway, stimulating tumor cell proliferation and enhancing their survival within the tumor microenvironment [27,28]. Disruption of the p53 pathway, a key tumor suppressor in cancer prevention, is commonly observed in CRC patients and can be influenced by toxic metabolites produced by an altered microbiome [31]. This interaction between pathogenic microorganisms and cellular pathways plays a crucial role in the neoplastic transformation of intestinal epithelial cells [31].

### 2.6. Dysbiosis as a Cause and Consequence of CRC

**Figure 1:**
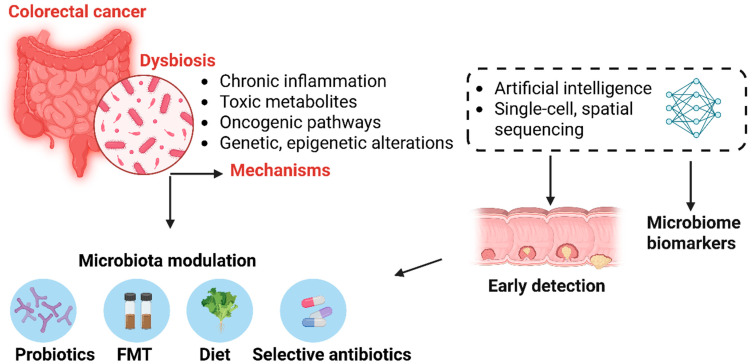
The role of gut microbiome dysbiosis in colorectal cancer pathogenesis and its potential for early detection and therapeutic intervention. Dysbiosis in the gut microbiota contributes to colorectal cancer (CRC) through several mechanisms, including chronic inflammation, production of toxic metabolites, activation of oncogenic signaling pathways, and induction of genetic and epigenetic alterations. Advances in single-cell and spatial sequencing, alongside artificial intelligence, enable the identification of microbiome—derived biomarkers for early CRC detection. Therapeutic modulation of the microbiota—via probiotics, fecal microbiota transplantation (FMT), dietary interventions, or selective antibiotics—offers promising strategies for CRC prevention and personalized treatment.

The imbalance of the gut microbiota is not only a consequence of CRC but also a determining factor in its pathogenesis [31,32]. Understanding the relationship between the microbiome and colorectal cancer provides new insights into carcinogenesis mechanisms and may pave the way for innovative prevention and treatment strategies (Figure 1). Modulating the gut microbiome through diet, probiotics, or targeted therapies against pathogenic microorganisms presents promising opportunities for CRC management [23].

A meticulous search of the literature was conducted using the PubMed and Google Scholar databases, based on a series of specific keywords designed to identify studies relevant to this work. The search included terms such as: “colorectal cancer”, “colorectal neoplasia”, “microbiome alterations”, “microbiome dysbiosis”, “microbiome disruption”, “therapeutic strategies”, “microbiome-targeted therapy”, and “microbiota modulation”.

To maintain a high level of relevance, the search parameters were intentionally restricted to studies specifically involving human subjects and publications available in the English language.

Many recent treatment strategies have been identified to modify gut microbiota to inhibit colorectal cancer (CRC) progression. Some of these strategies include probiotic and prebiotic therapy, fecal microbiota transplantation, selective antibiotic and antimicrobial treatment use, dietary modifications, and customized medication [33,34,35].

### 2.7. Probiotics and Prebiotics

Probiotics are live microorganisms that, when administered in appropriate amounts, confer health benefits by maintaining gut microbiota homeostasis and enhancing host immunity [36,37,38]. Numerous studies have demonstrated their capacity to suppress CRC cell proliferation, induce apoptosis, and modulate signaling pathways such as NF-κB and β-catenin, thus inhibiting tumor growth [38]. Besides their direct antitumor effects, probiotics play a pivotal role in strengthening the intestinal epithelial barrier, producing short-chain fatty acids (SCFAs) like butyrate, and competing with pathogenic bacteria for adhesion sites, reducing their colonization potential [26].

Prebiotics, on the other hand, are non-digestible food components that selectively stimulate the growth and activity of beneficial bacteria, including Bifidobacterium and Lactobacillus species, promoting a balanced microbial ecosystem [37]. Emerging formulations now combine prebiotics with specific probiotic strains in so-called synbiotics to maximize their synergistic effects in CRC prevention and therapy.

Clinical trials are underway to determine the optimal strains, dosages, and treatment durations, as well as to address safety concerns, especially in immunocompromised patients. Future research should aim to develop next-generation probiotics with tailored functions, potentially engineered to deliver anticancer molecules directly to the tumor microenvironment.

### 2.8. Fecal Microbiota Transplantation (FMT)

FMT involves the transfer of fecal matter from healthy donors into the gastrointestinal tract of CRC patients to restore microbiota diversity and composition [39,40]. It has gained recognition as an innovative tool capable of rebalancing dysbiotic gut environments, reducing inflammation, and modulating oncogenic pathways linked to tumorigenesis [41]. Preclinical and early-phase clinical studies indicate that FMT may enhance responses to chemotherapy and immunotherapy by modifying the tumor microenvironment and boosting anti-tumor immune activity [42].

Recent advances suggest that combining FMT with precision antibiotics or probiotics could further optimize its efficacy by selectively depleting harmful microbes while enriching beneficial taxa. Nevertheless, standardizing donor screening, stool preparation, and administration protocols remains a significant challenge, and concerns persist regarding the potential transmission of opportunistic pathogens and long-term microbiome shifts.

As research progresses, the integration of microbiota profiling and biomarker analysis could enable personalized FMT strategies, matching donors and recipients based on microbiome compatibility to maximize therapeutic benefit and minimize risks.

### 2.9. Antibiotics and Selective Antimicrobial Therapies

Traditional broad-spectrum antibiotics have been explored as a means to eradicate CRC-associated pathogens like *Fusobacterium nucleatum*, *Escherichia coli*, and *Bacteroides fragilis*, which are known to promote inflammation, genomic instability, and tumor progression [27]. While short-term antibiotic treatment can reduce bacterial load and alleviate inflammation, indiscriminate use disrupts the entire gut microbiota, potentially worsening dysbiosis and fostering antimicrobial resistance [43].

To overcome these drawbacks, novel approaches focus on narrow-spectrum antimicrobial agents and bacteriophage therapies that selectively target oncogenic bacteria without harming commensal species [26,43]. Additionally, antibiotic therapy may sensitize tumors to chemotherapeutic drugs by modulating microbial enzymes that influence drug metabolism.

Ongoing research is exploring synthetic antimicrobial peptides, CRISPR-based antimicrobials, and nanoparticle delivery systems as innovative means to enhance specificity and minimize collateral effects on the broader microbiome. These targeted strategies represent a promising frontier for integrating antimicrobial therapies into comprehensive CRC treatment regimens.

### 2.10. Diet and Nutraceuticals

Dietary patterns profoundly shape the gut microbiota composition and functional output [44,45,46]. Diets rich in fiber, resistant starches, and plant-derived polyphenols promote the growth of SCFA-producing bacteria, such as butyrate-producing *Faecalibacterium prausnitzii*, which exert anti-inflammatory and antineoplastic effects on the colonic mucosa [30,47]. Conversely, Western diets high in saturated fats, red meat, and processed foods encourage the expansion of bacteria that generate carcinogenic metabolites like secondary bile acids and nitrosamines [18,30].

Nutraceuticals—bioactive compounds derived from food sources—are gaining attention for their dual nutritional and therapeutic benefits. Omega-3 fatty acids, curcumin, and green tea polyphenols have demonstrated the ability to modulate gut microbiota composition, suppress pro-inflammatory pathways, and inhibit CRC cell proliferation in both preclinical and clinical settings [48,49].

Personalized dietary recommendations that consider individual microbiota profiles may enhance the preventive and therapeutic impact of nutrition in CRC management. Additionally, combining nutraceuticals with probiotics or prebiotics could provide synergistic benefits by reinforcing beneficial microbial communities and their metabolic functions.

### 2.11. Personalized Medicine and Future Directions in Microbiota-Based CRC Treatment

The era of precision oncology has catalyzed efforts to tailor CRC treatments based not only on genetic and molecular tumor characteristics but also on the patient’s unique microbiome signature [1,50]. Integrating microbiome data with genomic, transcriptomic, and metabolomic information allows for the design of highly individualized therapeutic strategies that consider host–microbe interactions [51].

Advanced computational models and artificial intelligence tools are increasingly used to identify microbiome-derived biomarkers predictive of treatment response or adverse effects, enabling real-time treatment optimization [52]. Furthermore, novel microbiome-editing techniques, such as engineered probiotics and designer bacteriophages, hold the potential to reprogram the gut ecosystem with unprecedented precision.

Future research should focus on large-scale, longitudinal cohort studies to validate predictive microbiome signatures and clarify causative links between specific microbes and treatment outcomes. Multidisciplinary collaborations will be crucial to translate these insights into clinical practice, overcoming regulatory and ethical barriers to implement microbiome-based interventions as a standard component of personalized CRC therapy. Table 1 provides a comparative overview of the main therapeutic strategies aimed at modulating the gut microbiome in colorectal cancer, highlighting their mechanisms of action, current clinical applications, benefits, and limitations.

Despite its potential, there are many challenges to overcome before gut microbiota modulation can be effectively integrated into cancer treatment. For such a complex microbiome, the mechanisms by which bacteria detect and process signals from the cancer microenvironment remain unclear. A deeper analysis of the gut microbiome and its interactions with the host, anticancer drugs, and other exogenous factors is necessary to improve the prognosis of CRC patients.

### 2.12. Integration of Emerging Technologies

Recent advances in biomedical research technologies are transforming our understanding of the gut microbiome’s role in CRC. Artificial Intelligence (AI) and machine learning algorithms have proven increasingly effective in analyzing complex, high-dimensional microbiome data. These tools enable the identification of microbial signatures predictive of CRC risk, prognosis, or therapeutic responsiveness, and they support the development of personalized interventions based on individual microbial profiles [52]. For instance, AI models have been trained to distinguish between healthy individuals and CRC patients based on metagenomic sequencing data with high accuracy, offering new diagnostic and prognostic capabilities.

In parallel, single-cell RNA sequencing (scRNA-seq) and spatial transcriptomics are beginning to elucidate how host cells and microbial communities interact at the cellular and tissue levels within the tumor microenvironment. These technologies allow for the mapping of immune and epithelial cell states in relation to microbial infiltration, revealing how specific bacteria may influence oncogenic signaling or immune evasion locally [53]. Moreover, spatial resolution techniques can uncover microbiota–tumor spatial colocalization patterns, which may have implications for targeted therapies [54,55].

Together, these emerging tools offer unprecedented insight into the dynamic and context-dependent interactions between the host and its microbiota in CRC, opening new avenues for precision medicine and integrative diagnostics [56].

## 3. Microbiome and Immunotherapy Outcomes

The human microbiome—especially the gut microbiota—has become recognized as a key regulator of host immune responses and a major factor influencing the success of cancer immunotherapy [57]. Over the past decade, pioneering research has shown that the gut microbiome not only shapes baseline immune activity but also affects the efficacy and the side-effect profile of immune checkpoint inhibitors (ICIs), such as anti-PD-1, anti-PD-L1, and anti-CTLA-4 antibodies [58]. Indeed, patients with a diverse and compositionally balanced gut microbiome generally experience more favorable clinical outcomes following treatment with ICIs. This beneficial microbial profile has been associated with enhanced antitumor immune responses, leading to longer progression-free survival and overall survival. Emerging evidence suggests that specific bacterial taxa—such as *Akkermansia muciniphila*, *Faecalibacterium prausnitzii*, and members of the *Ruminococcaceae* family—may contribute to this therapeutic advantage by promoting immune activation, modulating inflammatory pathways, and enhancing the efficacy of ICIs [59,60,61,62]. These beneficial microbial species are believed to enhance antitumor immunity through a variety of complementary mechanisms. They can promote the maturation and functionality of antigen-presenting cells (APCs), such as dendritic cells, thereby improving tumor antigen recognition and presentation. Additionally, they facilitate the infiltration and activation of effector T cells—particularly CD8^+^ cytotoxic T lymphocytes—within the tumor microenvironment, a critical step for effective tumor clearance. These microbes also influence the expression of co-stimulatory and co-inhibitory molecules on immune cells, helping to fine-tune immune activation and sustain antitumor responses [63].

Beyond its taxonomic composition, the functional potential of the gut microbiome—reflected in its collective gene expression profiles and metabolic activity—is increasingly recognized as a critical determinant of host immune modulation. Microbial-derived metabolites, including short-chain fatty acids (SCFAs) such as butyrate, acetate, and propionate, along with polyamines and secondary bile acids, exert multifaceted effects on key immunological processes. These include the differentiation and suppressive function of regulatory T cells (Tregs), the activation and antigen-presenting capacity of dendritic cells, and the maintenance of intestinal epithelial barrier integrity.

Among these, SCFAs are particularly notable for their context-dependent immunomodulatory roles. At physiological concentrations, they promote mucosal immune tolerance and support anti-inflammatory pathways, contributing to immune homeostasis. However, under specific pathological or therapeutic conditions, SCFAs may also augment systemic immune responses by enhancing T-cell effector functions and improving responsiveness to immune checkpoint inhibitors (ICIs).

**Figure 2:**
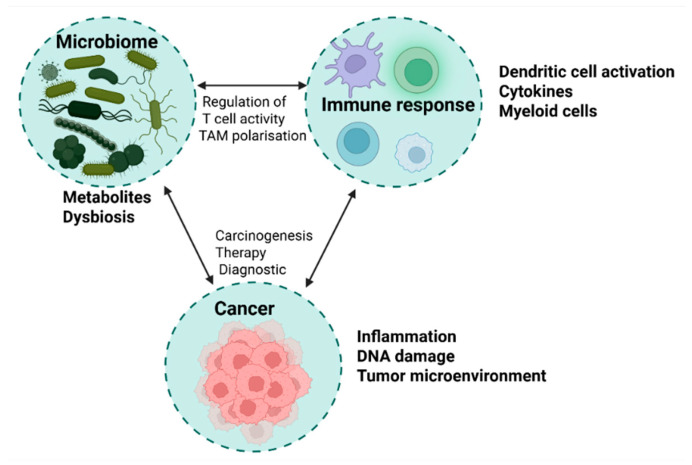
Microbiome–immune response–cancer axis: a triangular interaction model. This figure illustrates the dynamic interplay between the microbiome, immune response, and cancer development. On the left, the microbiome is represented by gut and tumor-associated bacteria, highlighting beneficial species (*Akkermansia*, *Faecalibacterium*, *Bifidobacterium*) and dysbiotic taxa (*Fusobacterium*, *H. pylori*). The immune response section (**top**) includes antigen-presenting cells, T cells, cytokines, and microbial metabolites (e.g., SCFAs, indoles) that modulate systemic immunity and tumor microenvironment activity. On the right, the cancer section depicts tumors influenced by chronic inflammation, immune evasion, and microbial-driven metabolic changes. Arrows indicate key signaling pathways, including microbial regulation of T-cell activity, tumor-associated macrophage polarization, and the impact of dysbiosis on carcinogenesis.

Moreover, alterations in microbiome composition and function have been correlated with systemic immune signatures observed in clinical responders to ICIs. Specifically, elevated circulating levels of pro-inflammatory cytokines such as interleukin-12 (IL-12), interferon-gamma (IFN-γ), and tumor necrosis factor-alpha (TNF-α) suggest that the gut microbiota may orchestrate a systemic immunostimulatory state that bridges intestinal and tumor immune compartments [64,65]. This emerging evidence underscores the microbiome’s capacity to act not merely as a passive bystander but as an active immunological intermediary, influencing the success of cancer immunotherapies (Figure 2).

In contrast, gut microbiome dysbiosis—defined by diminished microbial diversity, loss of beneficial commensal taxa, and the expansion of potentially pathogenic microorganisms (pathobionts) such as *Enterococcus faecalis* and *Escherichia coli*—has been consistently associated with suboptimal responses to ICI therapy [66]. This disrupted microbial ecosystem is thought to impair mucosal immune regulation and promote a pro-inflammatory milieu that undermines effective antitumor immunity. Clinically, such dysbiotic signatures have been linked to primary and acquired resistance to ICIs, shorter progression-free and overall survival, and an increased incidence of immune-related adverse events (irAEs). Notably, patients harboring dysbiotic microbiota profiles are more prone to develop severe inflammatory toxicities such as ICI-induced colitis and pneumonitis. [67,68]. These adverse effects are believed to result, in part, from microbiota-driven dysregulation of epithelial barrier integrity and aberrant activation of T-cell-mediated immune responses.

Emerging evidence also suggests that certain pathobionts may engage in direct immunomodulatory interactions—through pathogen-associated molecular patterns (PAMPs) or toxin production—that skew host immunity toward deleterious inflammatory pathways. These findings underscore the importance of maintaining microbial eubiosis not only for therapeutic efficacy but also for minimizing treatment-related complications in cancer immunotherapy.

## 4. Gut Microbiota Composition and Colorectal Cancer Subtypes

Recent evidence underscores that gut microbiota composition and functional capacity vary significantly across colorectal cancer subtypes, with important implications for tumor progression, immune landscape, and response to therapy. In particular, stratification of CRC based on the microsatellite instability status (MSI vs. MSS) and the Consensus Molecular Subtypes (CMS) framework reveals distinct microbial signatures that may influence treatment resistance [69,70,71].

MSI-high (MSI-H) tumors, which are characterized by defective DNA mismatch repair and high tumor mutational burden, often exhibit an inflamed tumor microenvironment enriched in immunostimulatory microbial taxa such as *Fusobacterium nucleatum*, *Bacteroides fragilis*, and *Peptostreptococcus anaerobius*. These microbial communities may support T-cell infiltration and antigen presentation, thus enhancing responsiveness to immune checkpoint inhibitors (ICIs). In contrast, microsatellite-stable (MSS) tumors typically show a less immunogenic phenotype and are associated with a dysbiotic microbiota marked by a higher abundance of pro-inflammatory and potentially oncogenic bacteria, contributing to a suppressive immune environment and resistance to ICIs [72].

Further stratification using the CMS classification provides additional insights into microbiota–cancer interactions [73]. CMS1, which overlaps with MSI-H tumors, is associated with high immune activation and microbial profiles rich in Bacteroides, Akkermansia, and short-chain fatty acid (SCFA)-producing species. This composition appears to synergize with immunotherapeutic responses. In contrast, CMS2 tumors, which are characterized by chromosomal instability and activation of WNT/MYC signaling pathways, often harbor reduced microbial diversity and increased levels of *Escherichia coli*, which have been implicated in inflammation and tumorigenesis. CMS2 tumors are generally resistant to ICIs and may also exhibit decreased responsiveness to fluoropyrimidine-based chemotherapy in the context of dysbiosis.

CMS3, the metabolic subtype, displays significant alterations in cellular metabolism and is often associated with microbiota enriched in *Lachnospiraceae* and *Enterococcus* spp., which may interfere with host metabolic pathways and contribute to chemoresistance. CMS4, the mesenchymal subtype, is associated with stromal invasion, TGF-β signaling, and poor prognosis. These tumors frequently exhibit a dysbiotic microbiota dominated by *Fusobacterium nucleatum*, which has been mechanistically linked to resistance to chemotherapeutics such as 5-fluorouracil and oxaliplatin [74]. This effect is mediated through the activation of TLR4 signaling, induction of autophagy, and suppression of apoptosis, contributing to treatment failure.

Beyond chemotherapy, microbiota-driven resistance to immunotherapy has been observed in MSS and CMS2/4 tumors, where a lower abundance of SCFA-producing bacteria and a dominance of pathobionts correlate with poor clinical outcomes. Microbial metabolites, immune signaling interference, and epithelial barrier disruption collectively modulate host–tumor–immune interactions, underscoring the relevance of microbiome profiling in predicting treatment response.

Altogether, these findings support the integration of microbiota analysis into molecular subtyping and therapeutic decision-making in CRC, with the goal of identifying microbial biomarkers of resistance and developing targeted microbiota-modulating interventions to improve patient outcomes.

## 5. Study Limitations and Future Directions

Although a growing body of evidence underscores the involvement of the gut microbiome in the pathogenesis and progression of colorectal cancer (CRC), several significant limitations continue to impede the translation of these insights into routine clinical practice. To date, much of the existing research remains predominantly observational, elucidating associations rather than establishing definitive causal links between gut dysbiosis and tumor development or progression. Moreover, substantial inter-individual variability in microbiome composition—shaped by dietary habits, antibiotic exposure, geographic and genetic factors, and underlying comorbidities—complicates the delineation of consistent microbial signatures that could serve as reliable diagnostic or prognostic biomarkers.

Furthermore, the complexity of host–microbe and microbe–microbe interactions, which are highly dynamic and context-dependent, further obscure mechanistic pathways. Additionally, the lack of standardized protocols for sample collection, sequencing methodologies, and bioinformatic analyses hinders reproducibility and limits cross-study comparability. Ethical and regulatory concerns, particularly regarding interventions such as fecal microbiota transplantation, also pose barriers to widespread clinical adoption.

To address these gaps, future research must prioritize large-scale, longitudinal, and mechanistic studies employing advanced metagenomic, metatranscriptomic, and metabolomic techniques to elucidate the precise pathways through which the microbiome modulates colorectal tumorigenesis. Integrating microbiome profiling with other multi-omics data (e.g., genomics, epigenomics, and metabolomics), combined with artificial intelligence and spatial analysis technologies, may yield deeper insights into the functional roles of microbial communities in CRC.

Equally important is the rigorous clinical evaluation of microbiome-targeted therapeutic strategies—including tailored probiotic and prebiotic formulations, dietary modifications, and standardized fecal microbiota transplantation protocols—to determine their safety, efficacy, and long-term impact on patient outcomes. Initiatives such as the Oncobiome project exemplify the progress made toward mapping cancer-specific microbial patterns and developing predictive and prognostic microbiome-based biomarkers. Ultimately, overcoming these challenges will be critical for integrating microbiome science into precision oncology and for realizing its full potential in enhancing CRC prevention, early detection, and treatment personalization.

## 6. Conclusions

Looking ahead, the integration of microbiome-based diagnostics and therapies into standard CRC treatment protocols holds great promise for enhancing patient survival and quality of life. Large-scale randomized clinical trials are essential to validate the safety, efficacy, and reproducibility of microbiome-targeted interventions. Furthermore, personalized microbiome profiling combined with other multi-omics data could enable precision oncology approaches tailored to each patient’s unique microbial and genetic background. Collaborative efforts among clinicians, microbiologists, and data scientists will be vital to overcome current limitations and translate scientific advances into real-world clinical benefits. As the field evolves, establishing clear regulatory frameworks and standardized methodologies will be critical to ensure the safe and effective application of microbiome-based innovations in colorectal cancer management.

## Figures and Tables

**Table 1:** Comparative overview of microbiome-targeted strategies in colorectal cancer treatment.

Therapy	Advantages	Limitations	Current Research Status
**Probiotics**	Restore gut microbial balance	Strain-specific effects	Promising in vitro and animal studies;
	Inhibit CRC cell growth	Individual response variability	Limited but growing clinical evidence
	Strengthen barrier—Reduce inflammation	Lack of standardized dosing	
**Prebiotics**	Promote beneficial bacteria	Effectiveness depends on baseline microbiota	Evidence mainly from dietary studies;
	Support SCFA production	Possible gastrointestinal side effects	Under investigation with probiotics
	Enhance probiotic effects		
**Fecal Microbiota Transplantation (FMT)**	Restore healthy microbial community	Risk of pathogen transmission	Emerging approach;
	Reduce inflammation	Lack of donor/procedure standardization	Some clinical studies show benefits in CRC associated dysbiosis; Needs more RCTs
	Modulate tumor microenvironment	Ethical/regulatory issues	
	Potential to overcome chemo resistance		
**Selective Antibiotics**	Target pathogenic CRC bacteria (e.g., *Fusobacterium*, *E. coli*, *B. fragilis*)	Risk of disrupting gut microbiota	Preclinical and early-phase studies;
	Reduce inflammation/tumor-promoting effects	Resistance risk	Research ongoing
		Long-term safety unclear	
